# Dysfunction of Nrf2-ARE Signaling Pathway: Potential Pathogenesis in the Development of Neurocognitive Impairment in Patients with Moderate to Severe Obstructive Sleep Apnea-Hypopnea Syndrome

**DOI:** 10.1155/2018/3529709

**Published:** 2018-08-12

**Authors:** Li Zhou, Ruoyun Ouyang, Hong Luo, Yating Peng, Ping Chen, Siying Ren, Guiqian Liu

**Affiliations:** ^1^Department of Pulmonary and Critical Care Medicine, The Second Xiangya Hospital, Central South University, 139 Renmin Middle Road, Changsha, Hunan 410011, China; ^2^Research Unit of Respiratory Disease, Central South University, 139 Renmin Middle Road, Changsha, Hunan 410011, China; ^3^Diagnosis and Treatment Center of Respiratory Disease, Central South University, 139 Renmin Middle Road, Changsha, Hunan 410011, China

## Abstract

The present study investigated the nuclear factor erythroid 2-related factor 2- (Nrf2-) antioxidant response element (ARE) signaling pathway in patients with moderate to severe obstructive sleep apnea-hypopnea syndrome (OSAHS). Their correlation with neurocognitive impairment metrics was investigated to explore potential pathogenesis in OSAHS. Forty-eight patients with OSAHS and 28 controls underwent testing with the Epworth Sleep Scale (ESS), MATRICS Consensus Cognitive Battery (MCCB), Stroop Color and Word Test, polysomnography (PSG), and measurements of the concentration of plasma superoxide dismutase (SOD) and thioredoxin (Trx). Further, 20 pairs of matched patients with OSAHS and controls were selected for measurement of the expression (protein and mRNA) of Nrf2 and of its downstream antioxidase, heme oxygenase-1 (HO-1), in peripheral mononuclear cells (PBMCs). Finally, correlations between neurocognitive impairment and the above metrics were analyzed. Expression of Nrf2 and HO-1 mRNA and protein in the PBMCs, as well as plasma SOD and Trx levels, were significantly reduced in patients with OSAHS. After adjusting for education, sex, age, and smoking index, the expression of Nrf2-ARE signaling pathway proteins (or mRNA) was closely correlated with sleep respiratory parameters. An inverse relationship was demonstrated between the expression of nuclear Nrf2 in PBMCs, concentration of plasma SOD and Trx, and apnea-hypopnea index (AHI) in patients with OSAHS. Trx, nuclear Nrf2 protein, and HO-1 protein were also negatively correlated with the percent of time that SaO_2_ was less than 90% (TSat90). Total Nrf2 protein level was positively correlated with AHI and TSat90 and negatively correlated with minimum SaO_2_ (LSaO_2_), while nuclear Nrf2 protein and HO-1 protein were positively correlated with LSaO_2_. Moreover, significant positive correlations were found between maze scores and expression of nuclear Nrf2 protein, HO-1 protein, and SOD and Trx levels. Furthermore, inverse relationships between total Nrf2 protein in PBMCs and HVLT-R and maze scores were found. Multiple linear regression showed plasma Trx concentration as a potential predictor of maze and BVMT-R scores. In conclusion, the expression of Nrf2-ARE molecules and related antioxidases is significantly decreased in patients with OSAHS and is correlated with neurocognitive dysfunction. The Nrf2-ARE signaling pathway may play a crucial role in neurocognitive impairment in patients with moderate to severe OSAHS. Further studies are needed to explore the exact mechanisms and potential treatment interventions.

## 1. Introduction

Obstructive sleep apnea-hypopnea syndrome (OSAHS) is characterized by nocturnal intermittent hypoxia/reoxygenation and sleep disturbances and is caused by repeated upper respiratory tract collapse during sleep. OSAHS has a roughly 22% prevalence in men and 17% in women, and this is higher in obese people and the elderly [1, 2]. Neurocognitive impairment is a common and vital clinical symptom in patients with OSAHS, though the exact physical and pathological mechanisms linking the two remain unclear. Oxidant-antioxidant imbalance has been considered as an important physiopathological response in patients with OSAHS [3]. Further, chronic intermittent hypoxia (CIH) would induce the accumulation of reactive oxygen species (ROS) and lead to dysfunction of mitochondria and the endoplasmic reticulum, which could then result in impaired ATP production, reduced antioxidant capacity, overproduction of proteins, DNA oxidation, lipid peroxidation, and dysfunction of cells and tissues. The brain, an organ with high oxygen consumption, is very vulnerable to hypoxia. Many studies have demonstrated that overproduction of oxidative stress products induced by CIH impair neurons and neural signaling pathways, and this likely plays a crucial role in the development of neurocognitive deficits in OSAHS [4]. However, studies exploring dysfunctional antioxidant signaling pathways in the development of neurocognitive impairment to find potential treatment strategies are lacking. Nuclear factor erythroid 2-related factor 2- (Nrf2-) antioxidant response element (ARE) is a vital signaling pathway that regulates the expression of several antioxidases in vivo. Nrf2 binds to Kelch-like ECH-associated protein 1 (KEAP1) in the cytoplasm and has low activity under normal physiological conditions. When cells are exposed to oxidative stress, Nrf2 uncouples with KEAP1, translocates into the nucleus, and binds to the ARE, thus initiating the transcription and expression of a series of downstream antioxidases such as heme oxygenase-1 (HO-1), superoxide dismutase (SOD), and thioredoxin (Trx), to maintain the body's physical balance [5]. It has been demonstrated that dysfunction of the Nrf2-ARE signaling pathway plays an important role in the development of cognitive decline in patients with neurodegenerative diseases [6, 7]. However, the role of the Nrf2-ARE signaling pathway in the development of neurocognitive impairment in patients with OSAHS has not been reported. Therefore, the present study sought to evaluate whether there are differences in the expression of Nrf2 mRNA and its downstream antioxidase, HO-1, in peripheral blood mononuclear cells (PBMCs) and related important antioxidases between patients with moderate to severe OSAHS and healthy controls. Further, we analyzed correlations between the Nrf2-ARE signaling pathway and neurocognitive impairment to explore the possible mechanisms of cognitive impairment seen in patients with OSAHS and to provide a foundation for further investigations. In addition, we found that there was no unified and relatively full-scale neurocognitive test battery to evaluate cognitive dysfunction in patients with OSAHS. The MATRICS Consensus Cognitive Battery (MCCB) [8] is an internationally recognized neuropsychological assessment test battery which includes the evaluation of executive function, attention, memory, and information processing speed which were the commonest cognitive impairment domains in OSAHS patients. The clinical reliability and validity of these tests have been examined in China and many other countries [9–11]. Thus, in the present study, we adopt MCCB to evaluate neurocognitive impairment in patients with moderate-severe OSAHS.

## 2. Materials and Methods

### 2.1. Participants

Forty-eight patients with moderate to severe OSAHS diagnosed using polysomnography (PSG) in the Second Xiangya Hospital of Central South University from September 2015 to March 2017 were included (41 men). Among them, 15 cases were moderate OSAHS and 33 were severe OSAHS. The control group consisted of 28 healthy volunteers (20 men) with no statistical differences from the OSAHS group in age, sex, body mass index (BMI), or years of education. The basic clinical characteristics of these two groups are listed in Table 1. We selected 20 matched pairs of OSAHS patients and controls (Table 2) for further analysis. This study was approved by the Ethics Committee of the Second Xiangya Hospital of Central South University. All subjects were informed of the study details and provided consent to participate.

Patients were included if they (1) had moderate to severe OSAHS, determined based on the guidelines recommended by the American Academy of Sleep Medicine (AASM; 2013 revision) [12], and (2) had not received any treatment for OSAHS. The exclusion criteria included (1) mild OSAHS; (2) BMI > 35 kg/m^2^; (3) history of brain, kidney, liver, blood, endocrine, or reproductive system diseases, malignant tumors, coronary heart disease, and so on; (4) current pregnancy; (5) use of hormones, hypnotics, sedatives, psychotropic substances, or tobacco; (6) infection, trauma, or major surgery in the previous 2 weeks; (7) history of any other respiratory diseases; (8) color blindness or color weakness; (9) previously received a similar or the same neuropsychological assessment; and (10) history of mental illnesses such as anxiety or depression. Where appropriate, these criteria applied to the healthy controls as well, except that they had no sleep disorder.

### 2.2. Daytime Sleepiness and Cognitive Function Assessment

Participants were provided with an Epworth Sleepiness Scale (ESS) evaluation and neuropsychological cognitive assessment (MCCB and Stroop Color and Word Test [13, 14], Table 3). These assessments were uniformly performed by a trained doctor in the sleep center. The doctor was blinded to the clinical information of every study participant before and after the assessment, and the order of evaluations was the same for every participant.

### 2.3. Polysomnography

All subjects received diagnostic PSG (Embla S4000; Medcare Technologies, Fuquay Varina, NC, USA). The monitoring was performed in a specialized sleep center.

### 2.4. Collection of Blood Samples

A 10 ml sample of fasted venous blood was collected from every participant and centrifuged at 3000 rpm for 5 min at 4°C. Plasma was removed and stored in a −80°C freezer. The PBMCs were separated from the remaining blood components via density gradient centrifugation and stored in three tubes. Two tubes were directly stored at −80°C for Western blot analysis, and 1 ml TRIzol (Thermo Fisher Scientific, Waltham, MA, USA) was added to the other tube for real-time quantitative PCR analysis, and the tube was stored at −80°C.

### 2.5. Detection of Plasma SOD and Trx Levels

Blood samples were analyzed using a commercially available sandwich enzyme-linked immunosorbent assay kit. The SOD testing kits were purchased from Abcam (Cambridge, UK) and the Trx testing kit was from Cusabio Biotech (Wuhan, China).

### 2.6. RNA Isolation and Quantitative Real Time PCR

Total PBMC RNA was extracted using TRIzol. The RNA concentration was measured using a spectrophotometer (Nanodrop 2000; Thermo Fisher Scientific), and first strand cDNA was generated using the RevertAid First Strand cDNA Synthesis Kit (Thermo Fisher Scientific). Relative expression levels of Nrf2 and HO-1 were measured using All-in-One qPCR Mix (GeneCopoeia, Rockville, MD, USA). Glyceraldehyde 3-phosphate dehydrogenase (*GAPDH*) was used as an internal control gene. The sequence of each primer was as follows:

*GAPDH*

Forward primer: 5′ GGAGCGAGATCCCTCCAAAAT 3′

Reverse primer: 5′ GGCTGTTGTCATACTTCTCATGG 3′

*Nrf2*

Forward primer: 5′ GCGACGGAAAGAGTATGAGC 3′

Reverse primer: 5′ TGGGAGTAGTTGGCAGATCC 3′

*HO-1*

Forward primer: 5′ ATGACACCAAGGACCAGAGC 3′

Reverse primer: 5′ GTGTAAGGACCCATCGGAGA 3′

Each cDNA sample was tested in triplicate. The 2-ΔCt method was used to calculate the relative expression levels of Nrf2 and HO-1 mRNA in the PBMCs of both the OSAHS group and the healthy control group (△Ct = target gene△Ct − internal control gene△Ct).

### 2.7. Western Blot Analysis

PBMCs were washed twice in ice-cold 1x phosphate buffered saline (PBS). Total protein was extracted with a protein lysate buffer (CWBio, Beijing, China), and nuclear proteins were extracted using a nuclear lysate buffer (Thermo Fisher Scientific). The protein concentration was determined using the bicinchoninic acid assay (BCA). After adjusting the concentrations, cell lysates were boiled at 100°C for 5 min. After cooling, each protein sample was separated on 10% Bis-Tris polyacrylamide gels and then electrotransferred to polyvinylidene difluoride (PVDF) membranes (MilliporeSigma, Burlington, MA, USA) via SDS-PAGE electrophoresis. The electrotransferred PVDF membranes were blocked in 5% skim milk dissolved in tris-buffered saline with 20% Tween-20 (TBS-T) for 1 h at room temperature and then incubated with specific antibodies against Nrf2 (ab137550, 1 : 1000; Abcam), HO-1 (SC-136960, 1 : 200; Santa Cruz Biotechnology, Dallas, TX, USA), *β*-actin (20536-1-AP, 1 : 2000; Proteintech, Rosemont, IL, USA), or proliferating cell nuclear antigen (PCNA; 10205-2-AP, 1 : 2000; Proteintech) overnight at 4°C. Membranes were then incubated with secondary antibodies labeled with horseradish peroxidase (HRP) for 1 h at room temperature. Finally, the membranes were imaged using chemiluminescence. *β*-Actin and PCNA were used as the internal control proteins to normalize slight variations in protein loading. The ratio of the optical density of the target protein and the internal control protein was taken as the relative expression of the protein.

### 2.8. Statistical Analysis

SPSS statistical software (version 23.0; IBM Corp., Armonk, NY, USA) was used for statistical analysis. Normally distributed data determined by a Kolmogorov-Smirnov test were analyzed using independent sample *t*-tests. Nonnormally distributed data were analyzed using nonparametric tests. Pearson's correlation coefficient was used for the correlation analysis of normally distributed data, and Spearman's rho was used for correlation analysis of nonnormally distributed data. Partial correlation analysis was used to analyze the correlation between levels of Nrf2-ARE signaling pathway, PSG parameters, and tests of neurocognitive function. Multiple linear regression was used to further evaluate the potential independent factors to predict the scores of neurocognitive function tests. A *p* value less than 0.05 was considered statistically significant.

## 3. Results

### 3.1. Neurocognitive Impairment

Differences in neurocognitive function between patients with OSAHS and healthy controls are shown in Table 4. Patients with OSAHS scored lower (*p* < 0.05) than healthy controls on all neurocognitive function tests included in MCCB and Stroop Color and Word Test (SCWT).

### 3.2. Nrf2-ARE Signaling Pathway Expression

**Figure 1:**
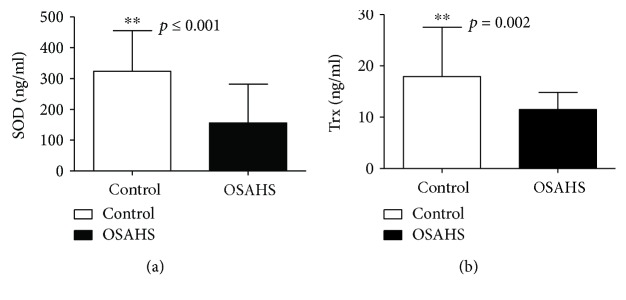
Plasma SOD and Trx level in OSAHS patients and healthy controls. (a) SOD levels for healthy controls and the OSAHS group, presented as mean ± SD. (b) Trx levels for healthy controls and the OSAHS group, presented as mean ± SD; ^∗∗^*p* < 0.01.

**Figure 2:**
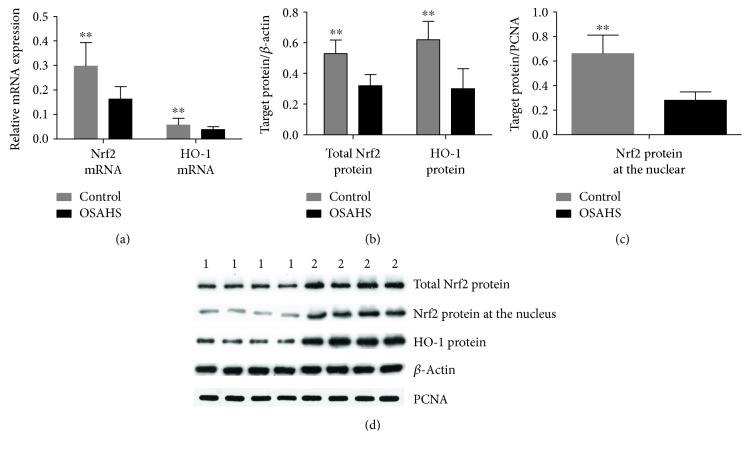
Nrf2 and HO-1 mRNA and protein expression in PBMCs of the OSAHS and control groups. (a) Relative mRNA expression of Nrf2 and HO-1 was analyzed by quantitative real-time PCR; normalized gene expression was obtained using the 2-ΔCt method. Data are expressed as mean ± SD; ^∗∗^*p*<0.01. (b-c) Relative protein expression of Nrf2 and HO-1 was analyzed using Western blot. Normalized protein expression was obtained as the ratio of optical density between target protein and *β*-actin. Data are expressed as mean ± SD. (d) The average relative quantification obtained by densitometric analysis of all the samples. ^∗∗^*p* < 0.01. 1—OSAHS group; 2—healthy control group.

Plasma SOD and Trx levels as well as Nrf2 and HO-1 mRNA and protein expression levels in PBMCs were significantly lower in the OSAHS group than in the control group (*p* < 0.01), as shown in Tables 5 and 6 and Figures 1 and 2.

### 3.3. Correlation between Sleep Breathing Parameters and Neurocognitive Function in Patients with OSAHS

**Figure 3:**
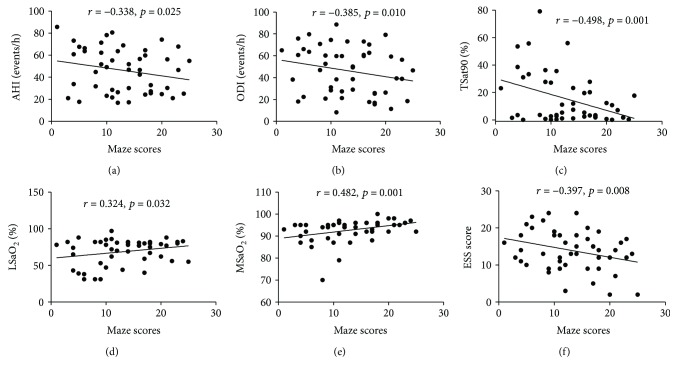
Correlation of NAB: maze scores with sleeping breathing parameters and ESS scores in the OSAHS group. NAB: maze scores are presented on the *x*-axis. PSG parameters are presented on the *y*-axis. AHI, apnea-hypopnea index; ODI, oxygen desaturation index; LSaO_2_, lowest oxygen saturation; MSaO_2_, mean oxygen saturation; TSat90, % of time SaO_2_ < 90%; REM%, percent of sleep spent in rapid eye movement phase; ESS, Epworth Sleepiness Scale.

**Figure 4:**
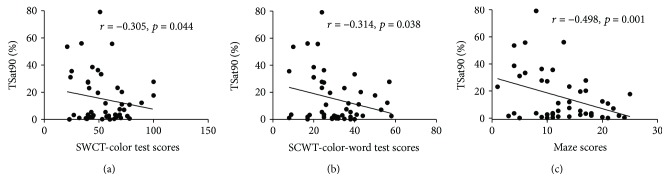
Correlation of TSat90 and neurocognitive function in the OSAHS group. After adjusting for sex, age, education and smoking index, TSat90 was correlated with SWCT test and maze scores.

**Figure 5:**
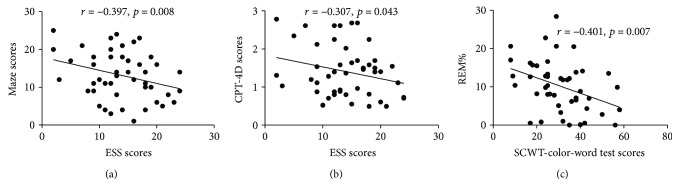
Correlation of ESS, REM%, and neurocognitive function in the OSAHS group. After adjusting for sex, age, education and smoking index, ESS scores were correlated with maze and CPT-4D scores and REM% was related with SCWT-color-word test scores.

Correlations between sleep breathing parameters and neurocognitive function in patients with OSAHS are presented in Table 7. In the OSAHS group, the NAB: maze scores were negatively correlated with AHI, ODI, % of time SaO_2_ < 90% (TSat90), and ESS scores, and positively correlated with LSaO_2_ and MSaO_2_ after adjusting for the influence of education, sex, age, and smoking index (Figure 3). TSat90 was also negatively correlated with the SCWT-color and SCWT-color-word test scores (Figure 4). The percent of rapid eye movement in total sleep time (REM%) showed negative correlation with SCWT-color-word test scores. Further, ESS scores were negatively associated with CPT-IP-4D scores (Figure 5).

### 3.4. Correlation between Sleep Breathing Parameters and Levels of Nrf2-ARE Signaling Pathway in Patients with OSAHS

**Figure 6:**
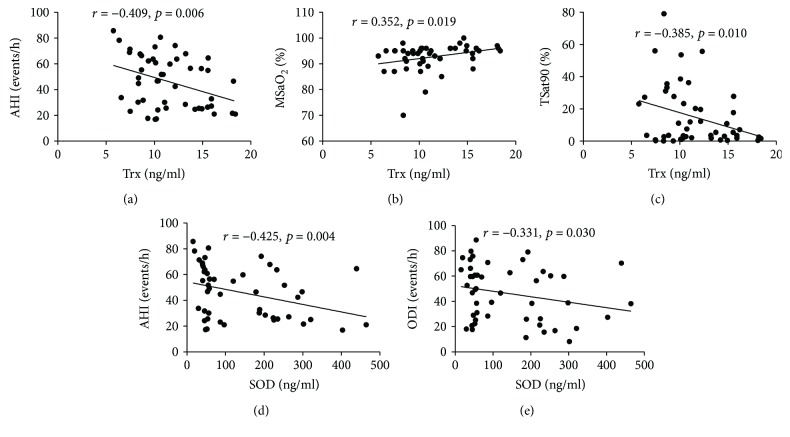
Correlation between plasma SOD and Trx and sleeping breathing parameters in the OSAHS group. Plasma Trx or SOD concentration are presented on the *x*-axis. Sleeping breathing parameters are shown on the *y*-axis. AHI, apnea-hypopnea index; ODI, oxygen desaturation index; LSaO_2_, lowest oxygen saturation; MSaO_2_, mean oxygen saturation; TSat90, % of time SaO_2_ < 90%; SOD, superoxide dismutase; Trx, thioredoxin.

**Figure 7:**
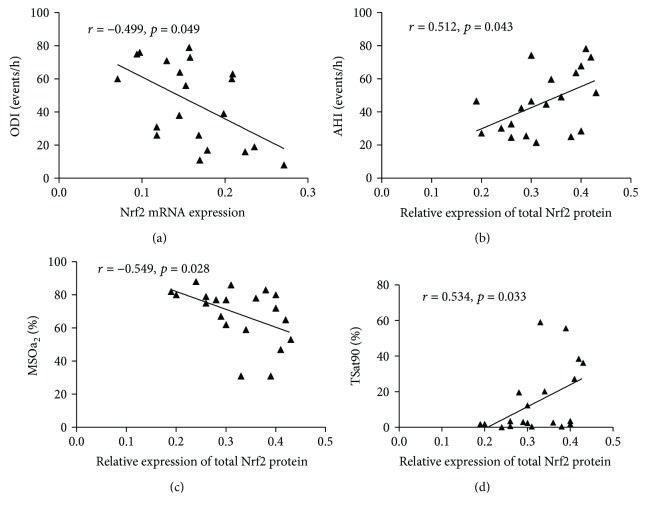
The correlation of Nrf2 mRNA and total Nrf2 protein expression in PBMCs and sleeping breathing parameters in the OSAHS group. AHI, apnea-hypopnea index; ODI, oxygen desaturation index; LSaO_2_, lowest oxygen saturation; MSaO_2_, mean oxygen saturation; TSat90, % of time SaO_2_ < 90%.

**Figure 8:**
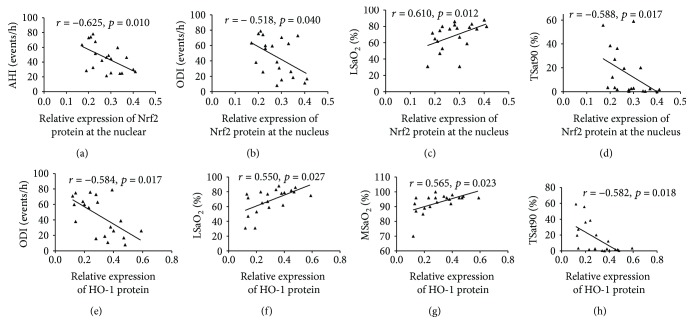
The correlation of relative expression of Nrf2 protein at the nucleus and HO-1 protein in PBMCs and sleeping breathing parameters in the OSAHS group. AHI, apnea-hypopnea index; ODI, oxygen desaturation index; LSaO_2_, lowest oxygen saturation; MSaO_2_, mean oxygen saturation; TSat90, % of time SaO_2_ < 90%.

As shown in Table 8, plasma SOD levels in the OSAHS group were negatively correlated with AHI and ODI after adjusting for the influence of education, sex, age, and smoking index (Figure 6). Moreover, plasma Trx levels were also negatively correlated with AHI and TSat90 and significantly positively correlated with MSaO_2_ (Figure 6). As shown in Table 9, Figures 7, and 8 after adjusting for the influence of education, sex, age, and smoking index, the expression level of Nrf2 mRNA in PBMCs in patients with OSAHS was negatively correlated with ODI. Expression of total Nrf2 protein in PBMCs in the same group was positively correlated with AHI and TSat90 and negatively correlated with LSaO_2_. However, nuclear Nrf2 protein expression in PBMCs was positively correlated with LSaO_2_ and negatively correlated with AHI, ODI, and TSat90. HO-1 protein expression was positively correlated with LSaO_2_ and MSaO_2_ and negatively correlated with ODI and TSat90. In addition, the expression of the above antioxidant indicators all showed no significant correlation with REM%.

### 3.5. Correlation between Nrf2-ARE Signaling Pathway and Neurocognitive Function in Patients with OSAHS

**Figure 9:**
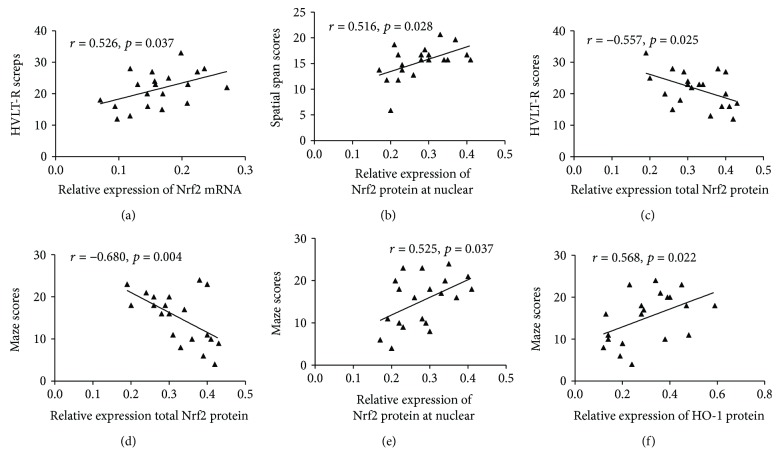
Correlations between Nrf2 and HO-1 mRNA or protein expression and neurocognitive function in the OSAHS group. HVLT-R, Hopkins Verbal Learning Test-Revised; WMS-III: spatial span, Wechsler Memory Scale-III: spatial span; NAB: mazes, Neurological Assessment Battery: mazes.

After adjusting for age, education, sex, and smoking index, plasma SOD and Trx levels were both positively correlated with performance on the NAB: maze test (Table 10). Further, plasma Trx levels were also positively correlated with scores on the HVLT-R, WMS-III: spatial span, BVMT-R, CPT-IP-2D, and SCWT tests (color and color-word). As shown in Table 11 and Figure 9, a positive correlation was shown between Nrf2 mRNA expression and scores on HVLT-R and WMS-III: spatial span tests. The expression level of total Nrf2 protein in PBMCs was negatively correlated with HVLT-R and NAB: maze scores. Furthermore, nuclear Nrf2 protein levels were significantly positively correlated with the performance on NAB: mazes and WMS-III: spatial span tests. HO-1 protein expression was also significantly positively correlated with the NAB: maze scores.

### 3.6. Prediction of Neurocognitive Function

A multiple linear regression was run to predict the results of neurocognitive function tests from independent variables including age, sex, smoking index, BMI, education, SOD, Trx, and PSG parameters. Education and plasma Trx concentration appeared to be statistically significant predictors of the BVMT-R scores (*F*(13, 34) = 3.367, *p* < 0.002, *R*^2^ = 0.563; Table 12). Age, educational years and plasma Trx concentration were also independent predictors of the NAB: maze score (*F*(11, 36) = 3.660, *p* < 0.002, *R*^2^ = 0.528) (Table 13). For the other neurocognitive tests, none of these variables were statistically significantly associated to scores of neurocognitive function tests.

## 4. Discussion

CIH in patients with OSAHS during sleep can produce large amounts of ROS, which might lead to oxidation/anti-oxidation imbalances, increased oxidative stress, and decreased antioxidant capacity—an important factor in the dysfunction of patients with OSAHS [3, 4]. Research has revealed that antioxidases such as aromatase, paraoxonase, sulfhydryl, and SOD, and antioxidants such as glutathione, vitamin A, and vitamin E are significantly lower in patients with OSAHS than in healthy controls [15–17]. This might reflect important pathophysiological mechanisms in the development of cardiovascular disease, renal damage, and neurocognitive dysfunction in these patients [3, 18]. The Nrf2-ARE signaling pathway is a crucial antioxidant signaling pathway that can activate downstream phase II antioxidant enzymes such as HO-1, SOD, Trx, catalase, NADPH: quinine oxidoreductase (NQO1), and glutathione peroxidase, among others, to maintain healthy function when under oxidative stress in cases like hypoxia, infection, inflammation, or injuries.

The Nrf2-ARE signaling pathway is dysfunctional in the mouse model of intermittent hypoxia (IH) [18–20]. The study found that the expression of Nrf2 and HO-1 were decreased in the kidneys of mice exposed to long-term (8 weeks) IH, while their expression was increased when exposed to short-term (3–7 d) IH. Moreover, metallothionein, an antioxidant protein, was also significantly decreased after long-term IH exposure [18]. Similarly, Wu et al. [19] found that wild-type mice with IH exposure for 3 d, 1 week, or 3 weeks showed increased expression of Nrf2 protein and mRNA as well as HO-1 and NQO1 mRNA expression in the kidney. However, the above indicators were all reduced when the mice were exposed to IH for 8 weeks. Further, the expression of Nrf2, HO-1, and NQO1 decreased in metallothionein knockout mice under both short-term and long-term IH treatment [19]. The levels of Nrf2 mRNA and protein in the genioglossus muscle of IH mice have also been shown to be reduced [20]. In vitro, HO-1 and Nrf2 expression was significantly decreased and the intracellular glutathione disulfide/glutathione ratio significantly increased after more than nine hours of IH in human aortic endothelial cells, while 8 h IH treatment upregulated the expression of intracellular Nrf2 and HO-1 [21]. When the IH mice received an HO-1 activator, IH-induced cellular oxidative stress and apoptosis could be alleviated [22].

In the present study, compared to those in the healthy control group, the mRNA and protein expression of Nrf2 and HO-1 in PBMCs as well as plasma levels of SOD and Trx in patients with OSAHS were all significantly reduced, meaning that antioxidant capabilities are systematically impaired in OSAHS. After adjusting for the influence of age, education, sex, and smoking index, the expression of Nrf2-ARE signaling pathway components in patients with OSAHS, including total Nrf2 protein, nuclear Nrf2 protein in PBMCs, and plasma Trx levels, were all correlated with TSat90 and AHI, and HO-1 protein levels were correlated with LSaO_2_, MSaO_2_, ODI, and TSat90. Furthermore, REM% showed no significant correlation with the above antioxidation indicators. Above results indicated that the impaired Nrf2-ARE signaling pathway may be more closely correlated to CIH, an important characteristic of OSAHS, rather than to disturbed sleep architecture. Furthermore, the expression of nuclear Nrf2 protein was negatively correlated with AHI and TSat90, while the expression of total Nrf2 protein was positively correlated with AHI and TSat90. This indicates that the balance of total Nrf2 protein and nuclear Nrf2 protein may be an important regulator of antioxidative capacity in OSAHS. In addition, levels of total Nrf2 protein, nuclear Nrf2 protein, SOD, and Trx were negatively correlated with AHI, which suggests that the Nrf2-ARE signaling pathway might also be closely associated with the severity of the disease.

Neurocognitive impairment, including declines in memory, attention, and executive function, is very common among patients with OSAHS. These impairments reduce the work efficiency and quality of life of patients with OSAHS to different degrees, in addition to negatively influencing social, public safety, and economic development on a societal level [1]. Disturbed sleep architecture and CIH are two major pathological factors in the development of neurocognitive impairment in OSAHS patients [23, 24]. Other factors include biochemical metabolic factors, genetic susceptibility, and comorbidities [24–26]. The brain, the organ with the highest oxygen consumption rate in the body and a high polyunsaturated fatty acid content, is extremely sensitive to hypoxia and ROS, especially in the cerebral cortex and hippocampus [27]. In OSAHS, CIH may cause mitochondrial dysfunction in the central nervous system, excessive activation of endoplasmic reticulum stress, production of large amounts of ROS/reactive nitrogen, excessive activation of oxidative stress, decompensation or downregulation of antioxidant capacity, and oxidation/antioxidant imbalance. Eventually, this could cause neuronal damage or apoptosis and abnormal signal transduction in the nervous system, which would gradually lead to neurocognitive impairment [3, 4, 28–30].

The Nrf2-ARE signaling pathway is one of the most important anti-oxidative signaling pathways in vivo. Studies have confirmed that Nrf2-ARE dysfunction plays a vital role in the development of cognitive dysfunction, especially in neurodegenerative diseases such as Alzheimer's disease (AD), amyotrophic lateral sclerosis, and Parkinson's disease [6]. Aggravated oxidative stress, mitochondrial dysfunction, and chronic neuroinflammation are the most common pathophysiological mechanisms in neurodegenerative diseases [7]. AD, with major clinical manifestations of progressive memory loss, cognitive dysfunction, and behavioral disorders, has a very close relationship with OSAHS. Approximately 30% of OSAHS patients have delayed-onset AD [31]. Men over 50 years of age with OSAHS have a 6-fold greater chance of developing AD compared to their peers [32]. Studies have shown that Nrf2 nuclear transcription is decreased and the Nrf2-ARE signaling pathway is deregulated in hippocampal neurons in patients with AD [33]. Further studies have demonstrated that patients with AD, characterized by beta amyloid deposition and numerous neuronal tangles, show decreased expression of Nrf2-ARE signaling pathway components, which promotes impaired synaptic plasticity and neuronal apoptosis damage, and then results in gradually declined and deteriorated memory and cognitive function [5].

Animal experiments have shown that sulforaphane, an Nrf2 activator, can reduce cerebral oxidative stress and inflammation, thereby improving cognitive impairment in an AD mouse model [34]. Both mRNA and protein expression of Nrf2 were also reduced in the motor cortex and spinal cord of patients with amyotrophic lateral sclerosis [35]. In patients with mild cognitive impairment and other neurodegenerative diseases, the expression of Nrf2-ARE signaling pathway components also decreases, which causes mitochondrial dysfunction, oxidative stress, inflammation upregulation, and downregulation of antioxidases and neuroprotective effects, before leading to cognitive impairment [6]. The regulation of endogenous signaling factors or exogenous Nrf2 activators can activate the Nrf2-ARE signaling pathway, upregulate the expression of downstream antioxidant enzymes such as HO-1, NQO1, and Trx, and improve mitochondrial dysfunction in the central nervous system. This could reduce oxidative stress and neuroinflammation, thus playing a neuroprotective role, which is an extremely valuable potential therapeutic target in the treatment of neurodegenerative diseases [5, 6, 36]. Li et al. [37] also demonstrated that hippocampal expression of HO-1 mRNA in CIH mice was increased during the first week and gradually decreased from the 8th day on. The present study shows that NAB: maze scores are positively correlated with total Nrf2 protein expression, nuclear Nrf2 protein, HO-1 protein in PBMCs, and plasma SOD and Trx levels in patients with OSAHS after adjusting for age, sex, education, and smoking index. Moreover, scores on HVLT-R and WMS-III: spatial span tests were correlated with Nrf2 mRNA, total Nrf2 protein, and plasma Trx levels. The multiple linear regression also showed that the Trx level was a potential predictor of NAB: maze and BVMT-R test scores. These results suggest that the Nrf2-ARE signaling pathway may play an important role in the decline of memory and executive function in patients with OSAHS. Further research is needed to explore the potential pathological mechanisms and treatment interventions, such as administration of Nrf2 activators, to improve and reverse the neurocognitive impairment in OSAHS. However, we did not observe a correlation between the expression levels of Nrf2 and test scores for attention or processing speed, indicating that Nrf2-ARE may be of little importance in the development of attention deficits in patients with OSAHS.

This study, however, is not without limitations. First, a low number of women were involved in this study due to the lower prevalence of OSAHS among the female population in China. In our sleep center, women account for fewer than 20% of patients with moderate to severe OSAHS. Moreover, obesity, a strong risk factor for OSAHS, is more common for middle-aged men in China. However, many studies have demonstrated a sex bias in physiological adaptations to oxidative stress in the healthy population as well as in patients with chronic neurodegenerative diseases, immune dysfunction, and cardiovascular disease, among others [38–40]. Men are more vulnerable to oxidative stress than women because of the role of testosterone; additionally, menopause and changes in estrogen level play an important role in oxidative stress levels in postmenopausal women [40]. Therefore, it is necessary to enroll equal proportions of men and women, or to recruit only either sex, to eliminate any sex bias. Second, the present study is a clinical observational study. We demonstrate a preliminary conclusion that the Nrf2-ARE signaling pathway may play a role in cognitive impairment in patients with OSAHS patients. However, the exact role of a disordered Nrf2-ARE signaling pathway in neurocognitive function among patients with OSAHS is uncertain. Ideally, a CIH mouse model should be established to further verify the detailed mechanisms. Third, the effects of drinking, socioeconomic status, physical activity, dietary factors, and occupational factors on antioxidation and neurocognitive impairment were not evaluated. These factors are also likely to influence the performance of neurocognitive tests to some extent. Fourth, considering that CIH may have little effect on neurocognitive function in patients with mild OSAHS, we only chose patients with moderate and severe OSAHS. That is, the result of this study did not apply to mild OSAHS patients.

## 5. Conclusions

The present study is the first to demonstrate that the expression of components of the Nrf2-ARE signaling pathway, an extremely crucial antioxidant pathway in vivo, and related key antioxidases in peripheral blood are all significantly decreased in patients with moderate and severe OSAHS. More importantly, the reduced expression of Nrf2-ARE proteins is significantly correlated with dysfunction of memory and executive function. Besides, we adopted MCCB, an internationally recognized battery of neurocognitive tests evaluating commonly impaired cognitive domains to evaluate cognitive impairment in patients with OSAHS. The results revealed that MCCB is likely to be a potentially effective neurocognitive test battery in assessing neurocognitive function in these patients. Moreover, we demonstrate that dysfunction of the Nrf2-ARE signaling pathway may be an important pathophysiological mechanism in the development of cognitive impairment in patients with moderate to severe OSAHS. The present study provides an important base for further research in vivo and in vitro to explore the specific mechanisms behind this dysfunction and potential treatments for improving neurocognitive function in patients with OSAHS.

## Figures and Tables

**Table 1:** Comparison of basic information between patients with OSAHS and healthy controls.

	Control group (*n* = 28)	OSAHS group (*n* = 48)	*P* value
Age (y)	40.8 ± 9.1	45.0 ± 10.1	0.070^a^
Men/women (*n*)	20/8	1/7	0.231^c^
Education (y)	15 (10–16)	12 (9–15)	0.119^b^
BMI (kg/m^2^)	27.24 ± 1.92	28.33 ± 3.36	0.494^a^
Smoking index	0 (0–350)	140 (0–400)	0.044^∗^^b^
Drinking index	0 (0–80)	0 (0–22.5)	0.928^b^
AHI (events/hour)	1.45 (0.65–2.4)	46.7 (25.8–63.7)	≤0.001^∗∗^^b^
LSaO_2_ (%)	93 (91–94)	77 (57–82)	≤0.001^∗∗^^b^
MSaO_2_ (%)	96 (96-97)	95 (91–96)	≤0.001^∗∗^^b^
ODI (events/hour)	1.2 (0.6–2.3)	48.1 (26–63.4)	≤0.001^∗∗^^b^
TSat90 (%)	0 (0–0)	5.4 (9.6–20.4)	≤0.001^∗∗^^b^
REM%	20.6 ± 2.69	10.2 ± 6.57	≤0.001^∗∗^^a^
ESS scores	9 (7–10)	14 (10–18)	≤0.001^∗∗^^b^

BMI: body mass index; ESS: Epworth Sleepiness Scale; TSat90, % of time SaO_2_ < 90%; REM%, percent of rapid eye movement in total sleep time. ^a^Student's *t*-test (data were presented as mean ± standard deviation (SD)). ^b^Mann–Whitney *U* test (data were presented as median (minimum-maximum)). ^c^Chi-squared test (data were presented as number of people). ^∗^*p* < 0.05 and ^∗∗^*p* < 0.01.

**Table 2:** Comparison of basic data between patients with OSAHS and healthy controls.

	Control group (*n* = 20)	OSAHS group (*n* = 20)	*P* value
Age (y)	41 (35–50)	48 (32–51)	0.301^b^
Men/women (*n*)	14/6	16/4	0.716^c^
Education (y)	13 (10–16)	12 (10–16)	0.429^b^
BMI (kg/m^2^)	27.8 ± 1.6	28.2 ± 3.6	0.626^a^
Smoking index	0 (0–0)	300 (0–675)	0.060^b^
Drinking index	0 (0–0)	0 (0–225)	0.640^b^
AHI (events/hour)	1.8 (0.8–2.4)	45.6 (27–63)	≤0.001^∗∗^^b^
ODI (events/hour)	1.0 (0.6–2.2)	47.6 (20–69)	≤0.001^∗∗^^b^
LSaO_2_ (%)	93 (91–94)	76 (60–80)	≤0.001^∗∗^^b^
MSaO_2_ (%)	96 (96-97)	96 (90–96)	0.024^∗^^b^
TSat90	0 (0–0)	3.25 (5.8–26.3)	≤0.001^∗∗^^b^
REM%	20.8 ± 2.97	9.05 ± 5.35	≤0.001^∗∗^^a^
ESS	8.5 (7–10)	13.5 (9.5–19)	≤0.001^∗∗^^b^

BMI: body mass index; ESS: Epworth Sleepiness Scale; TSat90, % of time SaO_2_ < 90%; REM%, percent of rapid eye movement in total sleep time. ^a^Student's *t*-test (data were presented as mean ± standard deviation (SD)). ^b^Mann–Whitney *U* test (data were presented as median (minimum-maximum)). ^c^Chi-square test (data were presented as number of people). ^∗^*p* < 0.05 and ^∗∗^*p* < 0.01.

**Table 3:** Description of MATRICS Consensus Cognitive Battery and SCWT.

Test	Cognitive domain	Variable of interest
TMT: part A	Speed of processing	Time to complete
Symbol coding	Speed of processing	Total number correct in 90 seconds
Category Fluency Test: animal naming	Speed of processing	Total number of animals named in 60 seconds
HVLT-R	Verbal learning (immediate memory)	Total number of words recalled correctly over three learning trials
WMS-III: spatial span	Working memory	Sum of raw scores on forward and backward conditions
NAB: mazes	Reasoning & problem solving	Total raw score
BVMT-R	Visual learning	Total recall score over three learning trials
CPT-IP	Attention/vigilance	The respective DPRIME values and average values for 2, 3, and 4 digits
SCWT	Executive function	The number of words, colors, and color words in 45 seconds

TMT: Trail Making Test; HVLT-R: Hopkins Verbal Learning Test-Revised; WMS-III: Wechsler Memory Scale-Third Edition; NAB: Neuropsychological Assessment Battery; BVMT-R: Brief Visuospatial Memory Test-Revised; CPT-IP: Continuous Performance Test-Identical Pairs; SCWT: Stroop Color and Word Test.

**Table 4:** Comparison of neurocognitive function between the control group and OSAHS group.

	Control group (*n* = 28)	OSAHS group (*n* = 48)	*P* value
TMT: part A	33.6 ± 9.06	44.3 ± 17.64	≤0.001^∗∗^^a^
Symbol coding	55.8 ± 14.6	44.1 ± 14.1	0.001^∗∗^^a^
HVLT-R	26 (21–30)	23 (16–27)	0.007^∗∗^^b^
WMS-III: spatial span	18 (16–19)	16 (13–17)	0.002^∗∗^^b^
NAB: mazes	18.2 ± 5.0	13.1 ± 6.2	≤0.001^∗∗^^a^
BVMT-R	22 (18–30)	20 (11–23)	0.002^∗∗^^b^
Category Fluency Test	23.9 ± 5.6	18.3 ± 6.2	≤0.001^∗∗^^a^
CPT-IP-2D	3.62 (3.17–4.24)	3.08 (1.95–3.68)	0.001^∗∗^^b^
CPT-IP-3D	3.18 (2.60–3.34)	2.34 (1.43–3.20)	0.009^∗∗^^b^
CPT-IP-4D	1.88 ± 0.79	1.40 ± 0.68	0.008^∗∗^^a^
CPT-IP-mean	2.76 ± 0.7	2.17 ± 0.8	0.002^∗∗^^a^
SCWT-word	95 (83–100)	80 (63–95)	0.003^∗∗^^b^
SCWT-color	69.4 ± 14.14	54.2 ± 19.1	≤0.001^∗∗^^a^
SCWT-color-word	41.8 ± 7.81	30.6 ± 12.5	≤0.001^∗∗^^a^

TMT: part A, Trail Making Test: part A; HVLT-R, Hopkins Verbal Learning Test-Revised; WMS-III: spatial span, Wechsler Memory Scale-III: spatial span; NAB: mazes, Neurological Assessment Battery: mazes; BVMT-R, Brief Visuospatial Memory Test-Revised; CPT-IP, Continuous Performance Test-Identical Pairs; SCWT, Stroop Color and Word Test; ^a^Student's *t*-test (data were presented as mean ± SD). ^b^Mann–Whitney *U* test (data were presented as median (minimum-maximum)). ^∗∗^*p* < 0.01.

**Table 5:** The difference of plasma SOD and Trx levels between the control and OSAHS groups.

	Control group (*n* = 28)	OSAHS group (*n* = 48)	*P* value
SOD (ng/ml)	312.7 (248.0–387.8)	78.2 (46.4–224.6)	≤0.001^∗∗^^b^
Trx (ng/ml)	17.93 ± 9.6	11.5 ± 3.32	0.002^∗∗^^a^

SOD, superoxide dismutase; Trx: thioredoxin. ^a^Student's *t*-test (data are presented as mean ± SD). ^b^Mann–Whitney *U* test (data are presented as median (minimum-maximum)). ^∗∗^*p* < 0.01.

**Table 6:** The expression difference of Nrf2 and HO-1 mRNA and protein between groups.

	Control group (*n* = 20)	OSAHS group (*n* = 20)	*P* value
Nrf2 mRNA	0.30 ± 0.097	0.16 ± 0.05	≤0.001^∗∗^^a^
HO-1 mRNA	0.050 (0.042–0.065)	0.039 (0.029–0.048)	0.004^∗∗^^b^
Total Nrf2 protein	0.53 ± 0.088	0.32 ± 0.073	≤ 0.001^∗∗^^a^
Nrf2 protein at the nucleus	0.66 ± 0.15	0.28 ± 0.07	≤0.001^∗∗^^a^
HO-1 protein	0.62 ± 0.12	0.30 ± 0.13	≤0.001^∗∗^^a^

Nrf2, nuclear factor erythroid 2-related factor 2; HO-1, heme oxygenase-1. ^a^Student's *t*-test (data are presented as mean ± SD). ^b^Mann–Whitney *U* test (data are presented as median (minimum-maximum)). ^∗∗^*p* < 0.01.

**Table 7:** Correlation between sleep breathing parameters and neurocognitive function test scores in the OSAHS group.

	AHI	ODI	LSaO_2_	MSaO_2_	TSat90 (%)	REM%	ESS
NAB: mazes	*r* = −0.338^∗^	−0.385^∗^	0.324^∗^	0.482^∗∗^	−0.498^∗∗^	0.099	−0.397^∗∗^
	*p* = 0.025	0.010	0.032	0.001	0.001	0.524	0.008
SCWT-color-word	*r* = 0.074	0.124	0.037	0.217	−0.314^∗^	−0.401^∗∗^	−0.130
	*p* = 0.632	0.422	0.809	0.157	0.038	0.007	0.402
SCWT-color	*r* = −0.042	0.069	0.175	0.194	−0.305^∗^	−0.142	−0.289
	*p* = 0.785	0.658	0.256	0.206	0.044	0.356	0.057
CTP-IP-4D	*r* = −0.110	0.011	−0.034	0.097	−0.056	−0.150	−0.307^∗^
	*p* = 0.478	0.941	0.825	0.531	0.717	0.330	0.043

AHI, apnea-hypopnea index; ODI, oxygen desaturation index; LSaO_2_, lowest oxygen saturation; MSaO_2_, mean oxygen saturation; TSat90, % of time SaO_2_ < 90%; REM%, percent of rapid eye movement in total sleep time; ESS, Epworth Sleepiness Scale; NAB: mazes, Neurological Assessment Battery: mazes; SCWT, Stroop Color and Word Test; CTP-IP, Continuous Performance Test-Identical Pairs. Data are presented as correlation coefficients (*r*) and *p* values from partial correlation analysis (adjusted covariates: age, education, sex, and smoking index). ^∗^*p* < 0.05 and ^∗∗^*p* < 0.01.

**Table 8:** Correlation between plasma SOD and Trx and sleeping breathing parameters in the OSAHS group.

*n* = 48	AHI	ODI	LSaO_2_	MSaO_2_	REM%	TSat90 (%)
SOD	*r* = −0.425^∗∗^	−0.331^∗^	0.133	0.299	0.078	−0.275
	*p* = 0.004	0.030	0.396	0.051	0.619	0.075
Trx	*r* = −0.409^∗∗^	−0.264	0.201	0.352^∗^	0.096	−0.385^∗^
	*p* = 0.006	0.083	0.190	0.019	0.537	0.010

Data are presented as correlation coefficients (*r*) and *p* values from partial correlation analysis (adjusted covariates: age, education, sex, BMI, and smoking index. ^∗^*p* < 0.05 and ^∗∗^*p* < 0.01. SOD, superoxide dismutase; Trx, thioredoxin; AHI, apnea-hypopnea index; ODI, oxygen desaturation index; LSaO_2_, lowest oxygen saturation; MSaO_2_, mean oxygen saturation; TSat90, % of time SaO_2_ < 90%; REM%, percent of rapid eye movement in total sleep time.

**Table 9:** Correlation of Nrf2 and HO-1 mRNA and protein expression in PBMCs and sleeping breathing parameters in the OSAHS group.

*n* = 20	Nrf2 mRNA	HO-1 mRNA	Total Nrf2 protein	Nuclear Nrf2 protein	HO-1 protein
AHI	*r* = −0.389	0.236	0.512^∗^	−0.625^∗^	−0.376
	*p* = 0.136	0.380	0.043	0.010	0.151
ODI	*r* = −0.499^∗^	0.269	0.486	−0.518^∗^	−0.584^∗^
	*p* = 0.049	0.313	0.057	0.040	0.017
LSaO_2_	*r* = 0.315	−0.265	−0.549^∗^	0.610^∗^	0.550^∗^
	*p* = 0.234	0.322	0.028	0.012	0.027
MSaO_2_	*r* = 0.430	−0.153	−0.439	0.424	0.565^∗^
	*p* = 0.096	0.573	0.089	0.102	0.023
REM%	*r* = 0.024	−0.147	0.202	0.265	0.026
	*p* = 0.931	0.586	0.454	0.321	0.924
TSat90 (%)	*r* = −0.418	0.139	0.534^∗^	−0.588^∗^	−0.582^∗^
	*p* = 0.108	0.609	0.033	0.017	0.018

Data are presented as correlation coefficients (*r*) and *p* values. Partial correlation analysis (adjusted covariates: age, education, sex, BMI, and smoking index. ^∗^*p* < 0.05. AHI, apnea-hypopnea index; ODI, oxygen desaturation index; LSaO_2_, lowest oxygen saturation; MSaO_2_, mean oxygen saturation; TSat90, % of time SaO_2_ < 90%; REM%, percent of rapid eye movement in total sleep time.

**Table 10:** Correlation of plasma SOD and Trx levels and neurocognitive function in the OSAHS group.

*n* = 48	Trx	SOD
HVLT-R	**r** = 0.352^∗^	0.223
	**p** = 0.019	0.150
WMS-III: spatial span	**r** = 0.378^∗^	0.186
	**p** = 0.011	0.233
NAB: mazes	**r** = 0.464^∗∗^	**0.349** ^∗^
	**p** = 0.002	**0.022**
BVMT-R	**r** = 0.461^∗∗^	0.211
	**p** = 0.002	0.175
CPT-IP-2D	**r** = 0.299^∗^	0.265
	**p** = 0.049	0.086
SCWT-color	**r** = 0.365^∗^	0.185
	**p** = 0.015	0.236
SCWT-color-word	**r** = 0.300^∗^	0.084
	**p** = 0.048	0.593

Data are presented as partial correlation coefficients (*r*) and *p* values. Partial correlation analysis (adjusted covariates: age, education, sex, BMI, and smoking index. ^∗^*p* < 0.05 and ^∗∗^*p* < 0.01. HVLT-R, Hopkins Verbal Learning Test-Revised; WMS-III: spatial span, Wechsler Memory Scale-III: spatial span; NAB: mazes, Neurological Assessment Battery: mazes; BVMT-R, Brief Visuospatial Memory Test-Revised; CPT-IP, Continuous Performance Test-Identical Pairs; SCWT, Stroop Color and Word Test.

**Table 11:** The correlation between Nrf2, HO-1 mRNA, protein expression, and neurocognitive function in the OSAHS group.

*n* = 20	Nrf2 mRNA	Total Nrf2 protein	Nuclear Nrf2 protein	HO-1 protein
HVLT-R	*r* = 0.526^∗^	**−0.557** ^∗^	0.460	0.243
	*p* = 0.037	**0.025**	0.073	0.364
WMS-III: spatial span	*r* = 0.581^∗^	−0.365	0.155	0.383
	*p* = 0.018	0.165	0.567	0.143
NAB: mazes	*r* = 0.446	**−0.680** ^∗∗^	**0.525** ^∗^	**0.568** ^∗^
	*p* = 0.083	**0.004**	**0.037**	**0.022**

Data are presented as partial correlation coefficients (*r*) and *p* values. Partial correlation analysis (adjusted covariates: age, education, sex, BMI, and smoking index. ^∗^*p* < 0.05 and ^∗∗^*p* < 0.01. HVLT-R, Hopkins Verbal Learning Test-Revised; WMS-III: spatial span, Wechsler Memory Scale-III: spatial span; NAB: mazes, Neurological Assessment Battery: mazes.

**Table 12:** Multiple linear regression of age, sex, BMI, education, smoking index, PSG parameters, and Trx and SOD levels for the prediction of BVMT-R test results.

Mode	Unstandardized coefficients		Standardized coefficients	*t*	Sig.	95.0% confidence interval for B	
	B	Std. error	Beta			Lower bound	Upper bound
1 constant	26.636	34.324		0.776	0.443	−43.119	96.390
Sex	−2.116	2.780	−0.104	−0.761	0.452	−7.765	3.534
Age (y)	−0.162	0.108	−0.226	−1.497	0.144	−0.382	0.058
Education (y)	0.779	0.236	0.420	3.308	0.002^∗∗^	0.301	1.258
Smoking index	0.003	0.002	0.175	1.204	0.237	−0.002	0.008
AHI	−0.084	0.089	−0.233	−0.949	0.350	−0.265	0.096
ODI	0.065	0.083	0.197	0.785	0.438	−0.104	0.235
LSaO_2_	−0.080	0.098	−0.191	−0.815	0.421	−0.279	0.119
MSaO_2_	−0.086	0306	−0.061	−0.281	0.780	−0.707	0.535
TSat90	−0.035	0.119	−0.088	−0.293	0.771	−0.227	0.207
REM%	−0.193	0.169	−0.174	−1.138	0.263	−0.537	0.151
BMI	−0.086	0.324	−0.40	−0.266	0.792	−0.745	0.572
Trx	0.996	0.376	0.454	2.65	0.012^∗^	0.232	1.760
SOD	−0.008	0.010	−0.139	−0.822	0.417	−0.029	0.012

Dependent variable: BVMT-R score. SOD, superoxide dismutase; Trx, thioredoxin; AHI, apnea-hypopnea index; ODI, oxygen desaturation index; LSaO_2_, lowest oxygen saturation; MSaO_2_, mean oxygen saturation; TSat90, % of time SaO_2_ < 90%; REM%, percent of rapid eye movement in total sleep time; BMI, body mass index; SOD, superoxide dismutase.

**Table 13:** Multiple linear regression of age, sex, education, smoking index, PSG parameters, and Trx levels to the prediction of the NAB: maze test scores.

Mode	Unstandardized coefficients		Standardized coefficients	*t*	Sig.	95.0% confidence interval for B	
	B	Std. error	Beta			Lower bound	Upper bound
1 constant	−10.86	27.328		−0.397	0.693	−66.285	44.564
Sex	3.027	2.313	0.174	1.309	0.199	−1.664	7.718
Age (y)	−0.221	0.091	−0.363	−2.446	0.019^∗^	−0.405	−0.038
Education (y)	0.579	0.201	0.366	2.882	0.007^∗∗^	0.171	0.986
Smoking index	0.002	0.002	0.132	0.994	0.351	−0.002	0.006
AHI	0.013	0.076	0.044	0.178	0.86	−0.14	0.167
ODI	−0.048	0.07	−0.17	−0.691	0.494	−0.189	0.093
LSaO_2_	0.018	0.084	0.052	0.22	0.827	−0.152	0.189
MSaO_2_	0.197	0.261	0.164	0.758	0.453	−0.331	0.726
TSat90	−0.032	0.102	−0.094	−0.31	0.768	−0.239	0.175
REM%	−0.056	0.144	−0.059	−0.389	0.699	−0.348	0.236
Trx	0.538	0.261	0.288	2.061	0.047^∗^	0.009	1.068

Dependent variable: NAB: maze score. Trx, thioredoxin; AHI, apnea-hypopnea index; ODI, oxygen desaturation index; LSaO_2_, lowest oxygen saturation; MSaO_2_, mean oxygen saturation; TSat90, % of time SaO_2_ < 90%; REM%, percent of rapid eye movement in total sleep time.

## Data Availability

The data used to support the findings of this study are available from the corresponding author upon reasonable request.
